# Preoperative chemoradiotherapy in rectal cancer induces changes in the expression of nuclear β-catenin: prognostic significance

**DOI:** 10.1186/1471-2407-14-192

**Published:** 2014-03-15

**Authors:** Jaime Gomez-Millan, Lydia Perez, Ines Aroca, Maria del Mar Delgado, Vanessa De Luque, Alicia Román, Esperanza Torres, Soraya Ramos, Sofia Perez, Eloisa Bayo, Jose Antonio Medina

**Affiliations:** 1 Department of Radiation Oncology, University Hospital Virgen de la Victoria, Campus Teatinos s/n, Málaga, 29010, Spain; 2 Department of Pathology, University Hospital Virgen de la Victoria, Malaga, Spain; 3 Centro de Investigaciones Biomedicas, Granada, Spain; 4 Department of Radiation Oncology, Hospital Juan Ramon Jimenez, Huelva, Spain; 5 Department of Medical Oncology, University Hospital Virgen de la Victoria, Malaga, Spain; 6 Department of Pathology, Hospital Juan Ramon Jimenez, Huelva, Spain

**Keywords:** Locally advanced rectal cancer, Radiotherapy, Chemotherapy, β-catenin

## Abstract

**Background:**

Preoperative chemoradiotherapy (CRT) is the cornerstone of treatment for locally advanced rectal cancer (LARC). Although high local control is achieved, overall rates of distant control remain suboptimal. Colorectal carcinogenesis is associated with critical alterations of the Wnt/β-catenin pathway involved in proliferation and survival. The aim of this study was to assess whether CRT induces changes in the expression of β-catenin/E-cadherin, and to determine whether these changes are associated with survival.

**Methods:**

The Immunohistochemical expression of nuclear β-catenin and membranous E-cadherin was prospectively analysed in tumour blocks from 98 stage II/III rectal cancer patients treated with preoperative CRT. Tumour samples were collected before and after CRT treatment. All patients were treated with pelvic RT (46–50 Gy in 2 Gy fractions) and 5-fluorouracil (5FU) intravenous infusion (225 mg/m^2^) or capecitabine (825 mg/m^2^) during RT treatment, followed by total mesorectal excision (TME). Disease-free survival (DFS) was analysed using the Kaplan-Meier method and a multivariate Cox regression model was employed for the Multivariate analysis.

**Results:**

CRT induced significant changes in the expression of nuclear β-catenin (49% of patients presented an increased expression after CRT, 17% a decreased expression and 34% no changes; p = 0.001). After a median follow-up of 25 months, patients that overexpressed nuclear β-catenin after CRT showed poor survival compared with patients that experienced a decrease in nuclear β-catenin expression (3-year DFS 92% vs. 43%, HR 0.17; 95% CI 0.03 to 0.8; p = 0.02). In the multivariate analysis for DFS, increased nuclear β-catenin expression after CRT almost reached the cut-off for significance (p = 0.06).

**Conclusions:**

In our study, preoperative CRT for LARC induced significant changes in nuclear β-catenin expression, which had a major impact on survival. Finding a way to decrease CRT resistance would significantly improve LARC patient survival.

## Background

Preoperative chemoradiotherapy (CRT) is the standard treatment for locally advanced rectal cancer (LARC). However, although high local control is achieved with multi-modality treatment, overall rates of distant control remain suboptimal in 30% of patients, and it is considered the leading cause of treatment failure [1].

Nowadays, molecular pathways of tumour resistance in rectal cancer are not fully understood and research focused on these mechanisms is urgently needed to improve patient survival. Colorectal carcinogenesis is associated with critical alterations of the Wnt/β-catenin signalling pathway [2]. β-catenin is a key multifunctional adaptor protein harbouring functions that are related to the subcellular location [3]. In the cytoplasm and within the membrane, β-catenin binds to intracellular E-cadherin and plays a role in cell adhesion and maintenance of normal cellular architecture. In the nucleus, β-catenin associates with members of the TCF-LEF family of transcription factors and activates the expression of target genes that enhance proliferation and cell survival. β-catenin is controlled by a multi-protein degradation complex, which contains the tumour suppressor adenomatous polyposis coli (APC), Axin, glycogen synthase kinase 3β (GSK3β) and casein kinase I [2,4].

Mutations occur in APC as an early event in the carcinogenesis of colorectal cancer, which results in an accumulation of β-catenin in the cytoplasm and translocation of β-catenin to the nucleus. Nuclear β-catenin binds to transcription factors of the high-mobility-group (HMG) box TCF/LEF family and results in enhanced proliferation and survival. β-catenin forms an adherens complex with E-cadherin, which is regulated by tyrosine phosphorylation [5] and which dissociates β-catenin from the complex and causes the release of β-catenin into the cytoplasm [6].

The association between the expression of nuclear β-catenin and patient survival has been previously described; however, the conclusions vary dramatically. Lugli et al. studied more than 1000 colorectal tumours initially treated with surgery, showing that an increase in nuclear β-catenin and a loss of membranous E-cadherin expression were independent prognostic factors for poor survival [7]. However, other reports have shown that increased nuclear β-catenin confers an advantage in survival [8].

Radiation has been shown to induce different molecular changes in both cellular RNA and proteins, resulting in increased proliferation, migration and cell cancer invasiveness. These effects counteract cell death, rendering the tumour more aggressive and decreasing the efficacy of radiation [9]. Some studies relate radiation resistance and the Wnt/β-catenin pathway. A recent study with pancreatic tumour xenografts has shown that radiation might induce radiation resistance through the phosphorylation and inhibition of GS3KB and the subsequent translocation of β-catenin to the nucleus [10]. Despite these preclinical results, the induction of changes in nuclear β-catenin and E-cadherin expression after RT or CRT and the implications for prognosis remain undetermined in the clinical setting.

In the present study, we aimed to prospectively evaluate changes in the expression profile of β-catenin and E-cadherin after CRT and the impact on survival in LARC patients treated with combined RT and 5-fluorouracil based CT.

## Methods

### Patient data and eligibility

Between January 2008 and December 2010, 98 patients with stage II-III (T2-T4 and/or N1-N2) rectal adenocarcinoma who were candidates for preoperative RT combined with CT were prospectively recruited in two centres.

Pretreatment evaluation included a complete history and physical examination with a digital rectal examination, colonoscopy with biopsy, abdomen and pelvic scan, chest X-ray, and magnetic resonance image (MRI) of the pelvis. Additionally, in 40% of patients, an endorectal ultrasound was performed. All patients were treated according to the routine protocol with pelvic RT (46–50 Gy in 2 Gy fractions) and 5-fluorouracil (5FU) intravenous infusion (225 mg/m^2^) or capecitabine (825 mg/m^2^) during RT treatment, followed by total mesorectal excision (TME) 6 weeks after CRT treatment. Local response to CRT was pathologically staged using criteria described by Mandard et al. [11] based on tumour regression grade (TRG) as follows: grade 1: tumour with fibrosis without tumour cells; grade 2: predominant fibrosis with scarce tumour cells; grade 3: fibrosis with tumour cells inside; grade 4: tumour cells outside of the fibrotic area; and grade 5: no tumour cells. Due to the low number of patients enrolled in the study, TRG was divided into two groups: group 1 comprised TRG 1–2 (good response) while group 2 comprised patients with regression grades 3–5 (poor response). Regional response was measured according to the presence or absence of tumour cells in the lymph nodes of the surgical specimen. After surgery, patients were treated with adjuvant chemotherapy (5-FU: 4 cycles of 500 mg/m^2^ once a day for 5 days repeated every 21 days, or capecitabine: 4 cycles of 1250 mg/m^2^ every 12 h for 14 days).

After treatment, all patients underwent clinical examinations and imaging on a regular basis. Patients were assessed for the occurrence of local, distant relapse, and death.

### β-catenin and E-cadherin immunostaining

Tumour samples were collected during diagnosis (pre-CRT) and during surgery (post-CRT). Samples were embedded in paraffin for immunohistochemistry (IHC) and serial cross-sections of each tumour sample were cut and stained with hematoxylin and eosin (H&E). β-catenin and E-cadherin IHC was performed on formalin-fixed, paraffin-embedded (FFPE) tissue. For the qualitative detection of β-catenin (rat monoclonal antibody clone βcatenin-1) and E-cadherin (rat monoclonal antibody clone NCH-38) a Dako Autostainer (Dako, Copenhagen, Denmark) was used. E-cadherin and nuclear β-catenin were examined by staining consecutive sections of each sample.

To study the expression of these proteins before CRT, a number of endoscopic biopsies ranging between 5 and 10 per tumour were fixed in formalin and embedded in paraffin. To investigate the expression after CRT, different paraffin blocks were obtained. After H&E staining, the block with the most representative part of the tumour was selected. Thus, in every section, the central and peripheral parts of the tumour were considered in order to measure the protein expressions. Two colon cancer sections known to be β-catenin and E-cadherin positive were used as positive controls, and omission of primary antibody was used as the negative control. The expression of β-catenin and E-cadherin were semi-quantitatively evaluated independently by two different pathologists without knowledge of the clinical and pathological parameters of the patients.

**Figure 1:**
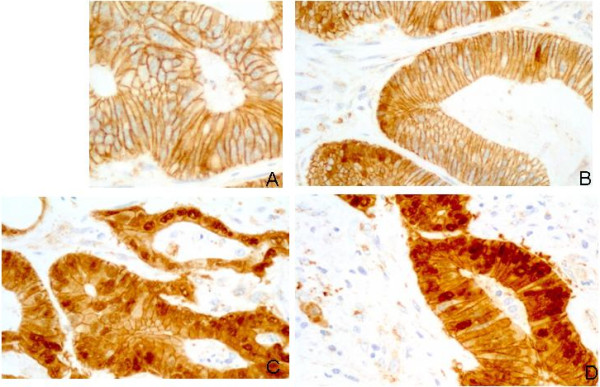
**Tumour cells showing different staining percentages for nuclear betacatenin. (A)**: Rectal cancer specimen showing absence of nuclear staining. **(B)**: low expression (less than 25% of cells). **(C)**: moderate expression (between 25% and 75% of cells). **(D)**: High expression (more than 75% of cells).

β-catenin expression in the nucleus was evaluated, and the percentage of tumour cells that expressed β-catenin was determined. We calculated the ratio between the number of tumour cells that expressed β-catenin and the whole number of tumour cells in the tissue section, before and after CRT (Figure 1). The expression was categorised as follows: absent (0% of cells); low (less than 25% of cells); moderate (between 25% and 75% of cells) or high (more than 75% of cells). For analytical purposes, the variable was dichotomised as low β-catenin expression (less than 25% of cells) and high β-catenin expression (25-100%) [12].

**Figure 2:**
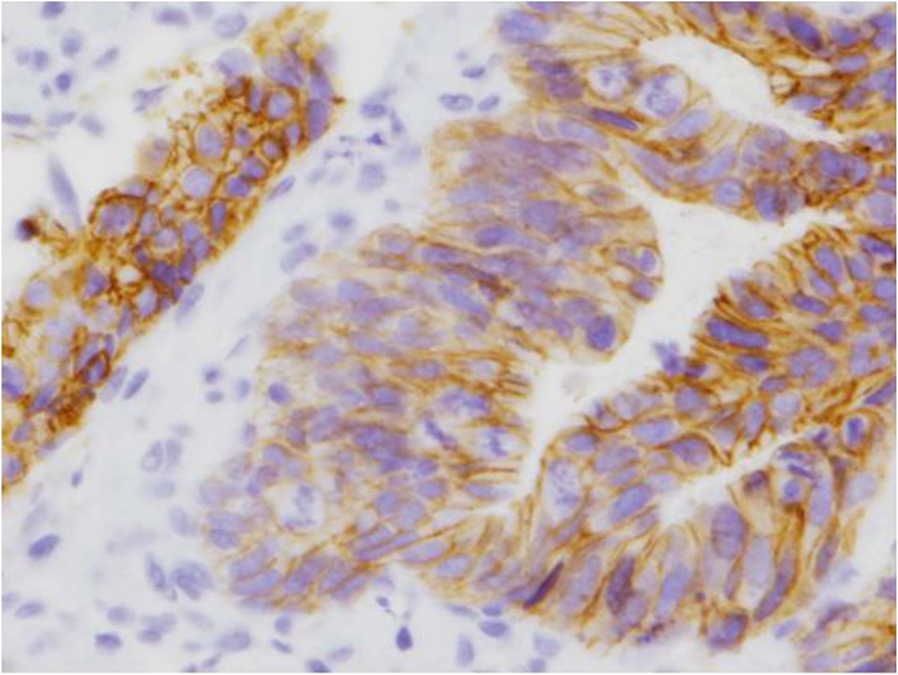
Rectal cancer specimen showing tumour cells with absence of membrane staining for E-cadherin.

E-cadherin expression in the membrane was evaluated based on the percentage of tumour cells that expressed E-cadherin (Figure 2). The expression was categorised as follows: absence (no expression); low (less than 25% of cells); moderate (between 25% and 75% of cells) or high (more than 75% of cells). E-cadherin expression was dichotomised based on absence (no expression) or presence (low, moderate and high expression) [7].

To ascertain the tumours that presented changes in the expression of β-catenin, we compared the expression levels of β-catenin before and after CRT. Changes in expression were categorised as follows: increase (from lack of expression to any other category, from low to moderate or high, and from moderate to high); decrease (from high to any category, from moderate to low or absence, and from low to absence); or equal (no change in category). To assess the differential expression of E-cadherin between pre-CRT and post-CRT samples, changes were categorised as follows: increase (from absence to any other category, from low to moderate, high, or no loss, and from moderate to high or no loss); decrease (from no loss to any other category, from high to moderate or low or absence, from moderate to low or absence and from low to absence) or equal (no change of category).

### Statistical analysis

Patients, type of treatment and disease characteristics were tabulated by means of frequency tables. Qualitative variables are expressed as a percentage with a 95 confidence interval of the percentage, and quantitative variables are expressed as the median and range. The association between qualitative dichotomised data of protein expression and clinico-pathological prognostic factors were compared using the chi-square test and Fisher’s exact test when appropriate. The Wilcoxon paired test was used for paired samples to compare pre-CRT and post-CRT protein expression levels. The end points of interest were tumour relapse and disease-free survival (DFS). DFS was defined as the time from first treatment to first documented relapse, secondary tumour or death by any cause. To investigate the pattern of occurrence over time of any of the aforementioned end points, descriptive analyses were carried out by estimating Kaplan-Meier survival curves, whereas inferential analyses relied on cumulative hazards. The threshold for significance for two-sided analysis was set to p > 0.05. Multivariate survival analysis was conducted using a multivariate Cox regression model. P values below the conventional 5% threshold were regarded as significant. All of the analyses were conducted using R and SPSS (Statistical Package for the Social Sciences) version 15.0 software.

### Ethics statement

This study was carried out in compliance with the Declaration of Helsinki ( http://www.wma.net/en/30publications/10policies/b3/index.html). All subjects provided informed consent for study inclusion, and the study was approved by our hospitals’ Ethics Committees (Comité de Etica of Hospital Virgen de la Victoria, Málaga, Spain; Comité de Etica of Hospital Juan Ramón Jiménez, Huelva, Spain).

## Results

### Clinico-pathologic characteristics of the patients

Of the 98 patients included, the vast majority were male, T3, with clinical lymph nodal metastasis and a distance ≤ 5 cm to anal verge. Tumours received a mean dose of RT of 47.9 Gy (range 46–50). After CRT, 44 patients (45%) presented a TRG 1–2, and 54 patients (55%) a TRG 3–5. After preoperative treatment, 28 patients (29%) presented lymph node metastases compared with 55 patients (56%) of lymph node metastases detected by imaging tests before CRT (P < 0.05). Table 1 describes the clinico-pathological data and distribution of the scores in the entire cohort of patients. A significant association was observed between the presence of a TRG 1–2 and the absence of lymph node metastasis in the surgical specimen (p = 0.01). Twenty-four patients (24%) presented a high expression of nuclear β-catenin (18 moderate and 6 high) and 46 patients (47%) presented an absence of E-cadherin expression in the membrane. There was neither an association between the absence of E-cadherin in the membrane and the expression of nuclear β-catenin (p = 0.4), nor significant associations between nuclear β-catenin or E-cadherin expression and clinico-pathological characteristics (Table 2).

**Table 1:** Clinico-pathological data and distribution of scores in the entire cohort of patients

		**E-cadherin**				**Nuclear β-catenin**			
**Factors**	**N (%) 98 (100)**	**Absent**	**Low**	**Mode-rate**	**High**	**Absent**	**Low**	**Mode-rate**	**High**
Sex									
M	72 (73%)	1 (1%)	2 (3%)	13 (18%)	56 (78%)	30 (42%)	26 (36%)	12 (17%)	4 (6%)
F	26 (27%)	1 (4%)	0 (0%)	5 (19%)	20 (77%)	9 (35%)	9 (35%)	6 (23%)	2 (8%)
Age									
</= 50	48 (49%)	2 (2%)	1 (1%)	15 (18%)	66 (77%)	33 (39%)	31 (37%)	15 (18%)	5 (6%)
>50	50 (51%)	0 (0%)	1 (7%)	3 (21%)	10 (72%)	6 (43%)	4 (29%)	3 (21%)	1 (7%)
Anal margin									
<= 5 cm	84 (86%)	1 (2%)	1 (2%)	7 (15%)	39 (81%)	21 (44%)	17 (35%)	7 (15%)	3 (6%)
>5 cm	4 (14%)	1 (2%)	1 (2%)	11 (22%)	37 (72%)	18 (36%)	18 (36%)	11 (22%)	3 (6%)
T stage									
T2-3	80 (82%)	2 (3%)	2 (3%)	15 (19%)	61 (85%)	29 (36%)	30 (37%)	15 (19%)	6 (8%)
T4	18 (18%)	0 (0%)	0 (0%)	3 (17%)	15 (83%)	10 (56%)	5 (28%)	3 (17%)	0 (0%)
N stage									
N-	43 (44%)	0 (0%)	1 (2%)	7 (13%)	44 (85%)	21 (40%)	16 (31%)	12 (23%)	3 (6%)
N+	55 (56%)	2 (4%)	1 (2%)	11 (24%)	32 (70%)	18 (39%)	19 (41%)	6 (13%)	3 (7%)
TGR*									
TGR 1	17 (17%)	0 (0%)	1 (6%)	3 (18%)	13 (76%)	5 (29%)	9 (53%)	2 (12%)	1 (6%)
TGR 2	27 (28%)	1 (4%)	0 (0%)	3 (11%)	23 (85%)	7 (26%)	11 (41%)	7 (26%)	2 (7%)
TGR 3	23 (24%)	1 (4%)	1 (4%)	2 (9%)	19 (83%)	11 (48%)	6 (26%)	4 (17%)	2 (9%)
TGR 4	26 (26%)	0 (0%)	0 (0%)	8 (31%)	18 (69%)	14 (54%)	7 (27%)	4 (15%)	1 (4%)
TGR 5	5 (5%)	0 (0%)	0 (0%)	2 (40%)	3 (60%)	8 (40%)	2 (40%)	1 (20%)	0 (0%)
pN ††									
pN-	70 (71%)	2 (3%)	1 (1%)	13 (19%)	54 (77%)	27 (39%)	26 (37%)	12 (17%)	5 (7%)
pN+	28 (29%)	0 (0%)	1 (4%)	5 (18%)	22 (78%)	12 (43%)	9 (32%)	6 (21%)	1 (4%)

*TGR: Tumor grade regression. †† pN: pathological lymphatic metastases.

**Table 2:** Distribution of nuclear β-catenin and E-cadherin relative to different clinicopathological prognostic factors

**Prognostic factors**	**All patients**		**E-cadherin**				**P**	**Nuclear β-catenin**				**P**
			**Absence vs. presence**					**Absence vs. presence**				
	**N**	**%**	**N**	**%**	**N**	**%**		**N**	**%**	**N**	**%**	
	**98**	**100**	**46**	**47%**	**52**	**53%**		**74**	**76%**	**24**	**24**	
Sex												
Male	72	73%	33	46%	39	54%	P=0.4	56	78%	16	22%	P=0.4
Female	26	27%	13	50%	13	50%		18	69%	8	31%	
Age												P=0.9
</= 50	48	49%	9	64%	36%	5	P=0.6	10	71%	4	29%	
>50	50	51%	37	44%	47	56%		64	76%	20	24%	
Distance to anal margin												P=0.7
</= 5 cm	84	86%	22	46%	26	54%	P=0.8	38	79%	10	21%	
>5 cm	14	14%	24	48%	26	52%		36	72%	14	28%	
T stage												P=0.4
T2-3	82	83%	38	47%	42	53%	P=0.8	59	74%	21	26%	
T4	17	17%	8	44%	10	56%		15	83%	3	17%	
N stage												P=0.2
N-	43	44%	19	43%	25	57%	P=0.5	19	43%	25	57%	
N+	55	56%	27	50%	37	50%		22	41%	32	59%	
TGR*												P=0.6
TGR 1-2	44	45%	18	41%	26	59%	P=0.2	32	73%	12	27%	
TGR 3-5	54	55%	28	52%	26	48%		42	78%	12	22%	
pN ††							P=0.8					P=0.9
pN-	70	71%	20	74%	7	26%		30	44%	41	56%	
pN+	28	29%	54	76%	17	24%		11	41%	16	59%	

*TGR: Tumor grade regression. †† pN: pathological lymphatic metastases.

### CRT induces changes in the expression of nuclear β-catenin

Of the 98 patients initially included in the study, a total of 69 were fully assessable in terms of availability of the tumoral specimen pre- and post-CRT. Twenty-nine patients were excluded from the analysis for several reasons: 17 patients presented a complete response (TRG 1), and 12 patients harboured only a few residual tumour cells that could not be assessed for IHC (5 patients with TRG 2 and 7 patients with unknown TRG). Clinico-pathological data of the 69 patients and distribution of the scores are described in Table 3.

**Table 3:** Clinico-pathological data and distribution of scores in the entire cohort of patients with pre- and post-CRT available specimens

		**E-cadherin**				**Nuclear β-catenin**			
**Factors**	**N (%) 69 (100)**	**Absent**	**Low**	**Mode-rate**	**High**	**Absent**	**Low**	**Mode-rate**	**High**
Sex									
M	50 (73%)	1 (2%)	1 (2%)	7 (14%)	41 (82%)	24 (48%)	14 (28%)	3 (16%)	3 (6%)
F	19 (27%)	1 (5%)	0 (0%)	4 (21%)	14 (74%)	9 (47%)	7 (37%)	9 (18%)	0 (0%)
Age									
</= 50	8 (12%)	0 (0%)	0 (0%)	2 (25%)	6 (75%)	4 (50%)	2 (25%)	1 (12%)	1 (13%)
>50	61 (88%)	2 (3%)	1 (2%)	9 (15%)	13 (21%)	29 (47%)	19 (31%)	11 (18%)	2 (3%)
Anal margin									
<= 5 cm	34 (49%)	1 (3%)	1 (3%)	5 (15%)	27 (79%)	18 (53%)	8 (23%)	5 (15%)	3 (9%)
>5 cm	35 (51%)	1 (3%)	0 (0%)	6 (17%)	28 (80%)	15 (43%)	13 (37%)	7 (20%)	0 (0%)
T stage									
T2-3	54 (79%)	2 (4%)	1 (2%)	8 (15%)	33 (80%)	24 (44%)	17 (32%)	10 (18%)	3 (6%)
T4	15 (22%)	0 (0%)	0 (0%)	3 (20%)	12 (80%)	9 (60%)	4 (27%)	2 (13%)	0 (0%)
N stage									
N-	37 (54%)	0 (0%)	1 (3%)	5 (13%)	31 (84%)	18 (49%)	9 (24%)	8 (22%)	2 (5%)
N+	32 (46%)	2 (6%)	0 (0%)	6 (20%)	24 (75%)	15 (47%)	12 (37%)	4 (13%)	1 (3%)
TGR *									
TGR 2	20 (29%)	1 (5%)	0 (0%)	2 (10%)	17 (85%)	7 (35%)	7 (35%)	5 (25%)	1 (5%)
TGR 3	20 (29%)	1 (5%)	1 (5%)	1 (5%)	17 (85%)	11 (55%)	5 (25%)	3 (15%)	1 (5%)
TGR 4	24 (26%)	0 (0%)	0 (0%)	6 (25%)	18 (75%)	13 (54%)	7 (29%)	3 (13%)	1 (4%)
TGR 5	5 (7%)	0 (0%)	0 (0%)	2 (40%)	3 (60%)	2 (40%)	2 (40%)	1 (20%)	0 (0%)
pN ††									
pN-	46 (67%)	0 (0%)	1 (4%)	2 (9%)	20 (87%)	22 (48%)	14 (30%)	8 (17%)	2 (4%)
pN+	23 (33%)	2 (4%)	0 (0%)	9 (20%)	35 (66%)	11 (49%)	7 (30%)	4 (17 %)	1 (4%)

* TGR: Tumor grade regression. †† pN: pathological lymphatic metastases.

Preoperative CRT significantly increased nuclear β-catenin expression (49% of patients presented increased expression after CRT, 17% decreased expression and 34% no change; p = 0.001). No significant changes in the expression of E-cadherin were observed after preoperative treatment (Table 4).

**Table 4:** β-catenin and E-cadherin expression before and after RT-CT

**β-catenin-E-cadherin expression**	**PRE RT-CT**		**POST RT-CT**		**P**
	**Patients**		**Patients**		
	**N (69)**	**%**	**N (69)**	**%**	
Nuclear β-catenin score					P=0.001
Absent	33	48%	23	33%	
Low	21	30%	19	28%	
Moderate	12	17%	15	22%	
High	3	4%	12	17%	
Membranous E-cadherin score					P=0.13
Absent	2	3%	5	7%	
Low	1	1%	1	1%	
Moderate	11	16%	8	12%	
High	16	23%	24	35%	
Very high	39	57%	31	50%	

β-catenin. Absent: Absence of cells expressing β-catenin; Low: Less than 25% of cells; Moderate: Between 25% and 75% of cells; High: More than 75% of cells. E-cadherin. Absent: Absence of cells expressing E-cadherin. Low: Less of 25% of cells expressing E-cadherin. Moderate: Between 25% and 75% of cells expressing E-cadherin. High: More than 75% of cells expressing E-cadherin. Very high: 100% of cells expressing E-cadherin.

### Recurrences

Among the 98 patients included in the initial cohort of the study, with a median follow-up time of 25 months (range 5–58), we observed 22 recurrences (23%): 6 (6%) locoregional failures and 16 (16%) distant failures (13 distant failures and 3 patients with distant and locoregional failure). No significant association was found between disease recurrence and nuclear β-catenin or E-cadherin expression at diagnosis (p = 0.4).

We analysed the pattern of recurrence in association with the increase or decrease of nuclear β-catenin expression after CRT. Of the 69 patients included, we observed 20 recurrences (29%): 5 (7%) locoregional failures and 15 (22%) distant failures (13 distant failures and 2 distant and locoregional failures). Interestingly, of the 20 patients with recurrent disease and available tumoral sample, 19 (95%) presented an increased nuclear expression after CRT. 100% of patients with metastatic disease presented an increase in nuclear β-catenin expression after CRT. On the other hand, considering all the patients that presented a decrease in the expression of nuclear β-catenin after CRT, 92% were free of disease at 3 years (p = 0.03).

### Survival

#### Effect of β-catenin and E-cadherin expression at diagnosis on patient survival

Of 98 patients initially included, with a median follow up of 25 months (range 5 to 58 months), 13 patients had died: 6 (46%) because of primary cancer and 7 (54%) for other causes. The 3-year OS and DFS rates were 90% and 78%, respectively.

**Figure 3:**
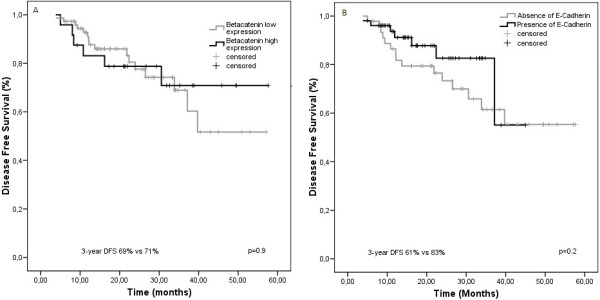
**Kaplan-Meier estimates of disease-free survival according to nuclear β-catenin and E-cadherin expression at diagnosis. A** and **B**. Data on disease-free survival (DFS) for the entire group are shown according to stratification on the basis of nuclear β-catenin expression and membranous E-cadherin expression.

No differences in survival were observed in patients with high nuclear β-catenin compared with those with low nuclear β-catenin (3 year DFS: 71% vs. 52%; HR = 0.93; 95% CI 0.37 to 2.4; p = .0.9) (Figure 3). Moreover, no survival differences were observed in patients with presence of E-cadherin compared with those with absence (3 year DFS: 71% vs. 52%; HR = 0.58; 95% CI 0.23 to 1.45; p = .0.2) (Figure 3).

#### Effects of changes of β-catenin and E-cadherin after RT-CT on survival

**Figure 4:**
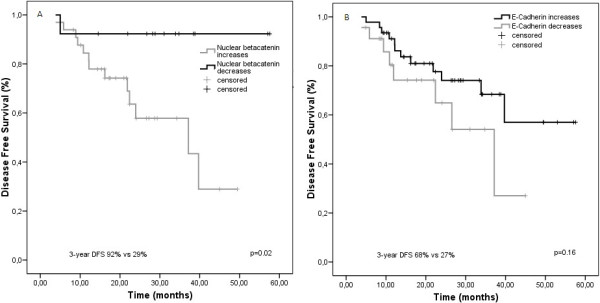
**Kaplan-Meier estimates of disease-free survival according to increase or decrease of β-catenin and E-cadherin expression after CRT. A** and **B**. Data on disease-free survival (DFS) for the entire group are shown according to stratification on the basis of the increase or decrease of nuclear β-catenin and membranous E-cadherin expression after CRT.

To study the effects of changes in β-catenin and E-cadherin expression on survival, we analysed the cohort of 69 patients with available samples pre- and post-CRT to consider whether an increased, decreased or equal expression of β-catenin and E-cadherin after CRT were associated with differences in disease-free survival rates. After preoperative CRT, changes in the expression of nuclear β-catenin were significantly associated with DFS rates. Patients with an increase in the number of cells that expressed nuclear β-catenin after CRT showed poor survival compared with patients who experienced a decrease (3-year DFS 92% vs. 43%, HR 0.17; 95% CI 0.03 to 0.8; p = 0.02) (Figure 4). However, patients with an increase in the number of cells with absence of expression of E-cadherin did not show a significant difference in survival (3-year DFS 69% vs. 27%, HR 1.8; 95% CI 0.8 to 4.7), compared with patients who experienced a decrease (Figure 4). In the Cox regression analysis with DFS as end point, when adjusting for N category, TRG and nuclear β-catenin expression, postoperative lymph node metastases and T stage were the only prognostic factors independently associated with a poor prognosis in the multivariate analysis. Increased nuclear β-catenin expression after CRT almost reached the cut-off for significance (p = 0.06) (Table 5).

**Table 5:** Multivariant Cox regression analysis

**Explanatory variable**	**Univariant**	**P**	**Multivariant**	**P**
	**HR (95% CI)**		**HR (95% CI)**	
Increase in β-catenin				0.06
Decrease vs. increase	0.14 (0.02-0.9)	0.02	0.13 ( 0.01-1.4)	
T stage				0.02
T4 vs. T2-T3	1.7 (0.7-4.3)	0.2	4 (1.2-13.4)	
N stage				0.6
N- vs. N+	0.4 (0.18.1.1)	0.07	0.7 (0.2-2.3)	
TGR*				0.4
3-5 vs. 1-2	3.6 (1.2-10.7)	0.01	0.5 (0.1-2.6)	
pN ††				0.01
pN - vs. pN+	0.3 (0.14-0.77)	0.007	0.2 (0.06-0.69)	

*TGR: Tumor grade regression. pN: pathological lymphatic metastases. †† pN: pathological lymphatic metastases.

## Discussion

Colorectal carcinogenesis is associated with critical alterations of the Wnt/β-catenin signalling pathway. In this prospective study, we found that preoperative CRT in rectal cancer significantly increased nuclear β-catenin expression in tumour cells, conferring a significantly higher risk of recurrence (p = 0.03) and a trend in poor survival compared with those who experienced decreased nuclear β-catenin expression after CRT (p =0.06).

Although RT is a major modality in the treatment of cancer, little is known about the molecular changes induced by RT with or without CT. Radiation has been shown to induce different molecular mechanisms to counteract cell death, and several preclinical studies have shown that radiation may promote proliferation, migration and tumour cell invasiveness, which could offset the therapeutic effects of radiation [9]. β-catenin is controlled by a multi-protein degradation complex, which contains the tumour suppressor APC, Axin, GSK3β and casein kinase I [2,4]. Mutation in the multi-protein degradation complex containing APC, resulting in β-catenin translocation to the nucleus [2,4] has been identified as one of the most important molecular events associated with colorectal carcinogenesis. It has been shown that radiation induces phosphorylation of GSK3β, an effect known to inhibit GSK3β kinase activity, resulting in β-catenin translocation to the nucleus [13]. Furthermore, a recent preclinical report with a xenograft model of pancreatic cancer has shown that radiation promotes the phosphorylation of GSK3β at serine 9. This event promoted the translocation of β-catenin from the cytosol to the nucleus, which increased transcriptional activity of the Wnt/β-catenin pathway, leading to radiation resistance [10]. Other preclinical investigations have shown that radiation may enrich progenitor cells with an activated Wnt/β-catenin signalling pathway, which leads to the development of radiation resistance in breast cancer cells [14]. Finally, in head and neck cancer cell lines, radiation has been shown to induce the translocation of β-catenin to the nucleus, conferring radiation resistance through upregulation of Ku expression [15]. However, in the clinical setting, there are no published investigations that link radioresistance with the expression of nuclear β-catenin.

Our results have shown that preoperative CRT significantly increases nuclear β-catenin expression in tumour cells, which confers significantly poorer survival compared with those who experienced a decrease in nuclear β-catenin expression (p = 0.02). This finding almost reached the cut-off for significance in multivariate analysis (p = 0.06). Moreover, 93% of the patients who presented with recurrent disease also showed an increase in the expression of nuclear β-catenin (p = 0.03). On the other hand, patients who experimented a decrease of nuclear β-catenin expression after CRT showed an excellent prognosis, with 3 year DFS of 92% vs. 29% (HR 0.17; 95% CI 0.03 to 0.8; p = 0.02). To the best of our knowledge, this study provides the first clinical evidence to support the hypothesis that preoperative CRT in LARC increases nuclear β-catenin expression in tumour cells, which confers a significantly higher risk of recurrence and poor survival.

In accordance with other series, our results show that metastatic recurrence is the main pattern of recurrence for our patients and isolated locoregional recurrence occurs rarely after TME plus RT [1]. Nowadays, the leading cause of treatment failure in LARC treated with preoperative CRT is metastatic disease [1]. Thus, any improvement in the survival of these patients will require a better control of distant disease. The Wnt/β-catenin pathway stimulates expression of the target genes implicated in invasion, motility and proliferation [2]. Activation of this pathway as a result of CRT, with the consequent increase in the expression of nuclear β-catenin, may be a plausible mechanism of distant failure. Thus, this prognostic biomarker may potentially identify patients with a high risk of distant recurrence in which new adjuvant therapies targeting the Wnt/ β-catenin pathway might be investigated. However, this finding must be confirmed prospectively in clinical trials. One recent retrospective clinical study with 48 patients analysed the expression of β-catenin after preoperative CRT in rectal cancer patients, and no differences were found in nuclear β-catenin expression before or after CRT [16], although the limited sample size of this retrospective study may be considered as biased.

Previous prognostic data on nuclear β-catenin expression at diagnosis in colorectal cancer have shown conflicting results [7,8]. Our results have shown that patients with high levels of nuclear β-catenin at diagnosis do not have a significantly different DFS compared with those with low nuclear β-catenin expression. Other factors involved in this complex signalling pathway may play a hidden role that explains these non-significant differences in prognosis observed for basal nuclear β-catenin expression. Finally, β-catenin binds to intracellular E-cadherin and plays a leading role in cell adhesion and cellular architecture. Different authors have shown that the absence of membranous E-cadherin is independently associated with a poor survival rate in colorectal cancer treated with surgery upfront [17-19]. In contrast, the absence of E-cadherin was not a significant prognostic factor in our patients.

Our study implies some difficulties that should be mentioned. Characterising a tumour that has been treated with CRT is a challenge for several reasons: patients with TRG1 do not show residual tumour cells after CRT, and no tumoral tissue is available for the analysis. Furthermore, in some cases, preoperative CRT leads to histological changes with no gross tumour visible in the mucosa or a scarce number of cells that may make analysis difficult [20]. Moreover, the small size of the endoscopic biopsy taken in the diagnostic procedure should be considered as it may not be representative of the tumour studied. There are also certain difficulties derived from the lack of standardisation in the evaluation of β-catenin expression and the heterogeneity that most colorectal cancers have with respect to the distribution of nuclear β-catenin expression [21].

These factors render evaluation of the number of cells that harbour nuclear β-catenin difficult, hindering a comparison of the pre- and post-treatment expression of this protein in the same tumour. For all these reasons, our results should be taken with caution and should be confirmed with further studies.

However, some strengths of our study include the homogeneity of our treatment approach, the prospective design, and the assessment by two independent pathologists. Despite the limited sample size, the poor prognostic value of nuclear β-catenin after CRT reached statistical significance.

## Conclusions

In summary, our study provides the first evidence that preoperative CRT in LARC patients induces increased nuclear β-catenin expression in tumour cells and confers poor survival compared with patients who experience decreased nuclear β-catenin expression. Overexpression of nuclear β-catenin after CRT may help identify a subgroup of patients in whom adjuvant therapies may be tested for a better control of systemic disease and an improvement in survival.

## Abbreviations

LARC: Locally advanced rectal cancer; CRT: Chemoradiotherapy; RT: Radiotherapy; DFS: Disease-free survival; OS: Overall survival; IHC: Immunohistochemistry; TRG: Tumoral regression grade.

## Competing interest

The authors declare that they have no competing interest.

## Authors’ contributions

JG contributed with the concept, design and draft of the manuscript. IA, LP, MD, SRG, VD, SP contributed with acquisition and analysis of data. AR, ET, JM, EG contributed with the draft of the manuscript. All authors have read and approved the final manuscript.

## Pre-publication history

The pre-publication history for this paper can be accessed here:

http://www.biomedcentral.com/1471-2407/14/192/prepub
